# The dual-continua model of mental health in daily life: momentary associations between distress and wellbeing in clinical and non-clinical populations

**DOI:** 10.1016/j.jad.2025.119432

**Published:** 2025-05-19

**Authors:** Jackson Mason-Stephens, Tanika R. Sgherza, Kenneth G. DeMarree, Kristin Naragon-Gainey

**Affiliations:** a School of Psychological Science, University of Western Australia, Australia; b Department of Psychology, University at Buffalo, the State University of New York, United States of America

**Keywords:** Dual-continua model of mental health, Depression, Anxiety, Wellbeing, Clinical populations, Ecological momentary assessment

## Abstract

**Background:**

The dual-continua model suggests that psychological distress and mental wellbeing are related, yet distinct constructs. Previous studies have supported the model in general populations, though they have relied almost entirely on retrospective trait measures. No studies have contrasted clinical and non-clinical populations to investigate the association between distress and wellbeing as momentary experiences in daily life.

**Methods:**

This study used ecological momentary assessment (42 reports over 7 days) and multilevel structural equation modelling to analyse the strength of the momentary associations between distress and wellbeing (hedonic and eudaimonic) in participants with and without a clinical disorder (N = 345). We hypothesised that clinical diagnostic status (any diagnosis, anxiety diagnosis, or depression diagnosis) would predict a stronger negative within-person association between distress and wellbeing.

**Results:**

The momentary association between distress and wellbeing was strong and negative for hedonic well-being, and moderate and negative for eudaimonic wellbeing. Contrary to hypotheses, only depression diagnosis predicted the strength of the momentary distress-wellbeing association, wherein participants with depression tended to have a *weaker* negative association of distress with both types of wellbeing.

**Conclusion:**

Distress and wellbeing appear to be related but distinct momentary experiences in daily life regardless of one’s clinical diagnostic status, suggesting that each is important to consider in clinical settings.

## 1. Introduction

Historically, conceptualisations of mental health generally followed a medical model whereby mental illness and wellbeing form a single bipolar continuum (Keyes, 2005), and individuals are categorised as either mentally ill or healthy (Keyes, 2007; Westerhof and Keyes, 2010). The *dual-continua model of mental health* refutes this bipolar conceptualisation, instead suggesting that psychological distress (e.g., symptoms of depression or anxiety) and mental wellbeing (e.g., positive feelings and positive life functioning) operate as related, yet distinct constructs (Greenspoon and Saklofske, 2001; Keyes, 2002, 2009). Importantly, the dual-continua model allows for the identification of mental health profiles that are not possible in bipolar conceptualisations: for example, people may experience high wellbeing along with high distress (Keyes, 2005, 2009).

Empirical evidence for the dual-continua model has been well-established in general populations varying in age and cultural context (Keyes et al., 2008; Lamers et al., 2011; Mason Stephens et al., 2023; Westerhof and Keyes, 2010). Specifically, structural analyses indicate that wellbeing and distress load on two distinct factors (Iasiello et al., 2020). Furthermore, measures of distress and wellbeing differentially predict, and are differentially predicted by, variables such as health-risk behaviour (Venning et al., 2013), personality (Spinhoven et al., 2015), and self-efficacy (Schönfeld et al., 2016). Together, these findings suggest that overall mental health requires independent consideration of both distress and wellbeing.

### 1.1. Dual-continua model in clinical populations

The utility of the dual-continua model in clinical populations (i.e., individuals diagnosed with a psychological disorder) is a source of debate, with some arguing that the presence of mental illness or severe distress may be incompatible with wellbeing (Huppert, 2005). There are numerous foundations for this theoretical position. The *dynamic model of affect* postulates that additional cognitive load induced by stressful life events may lead to undifferentiated affective judgments, thereby strengthening the negative correlation between self-reported positive and negative affect (Zautra et al., 2001). In other words, when distress severity surpasses a particular threshold, psychological dimensions of distress and wellbeing may become less distinguishable or even indistinguishable. Equally, the functional impairment in major life domains (e.g., loss of relationships or employment, or reduced participation in pleasurable/meaningful activities) associated with psychological disorders may partially or completely inhibit wellbeing-promoting experiences.

Furthermore, differences in the distress-wellbeing association might depend on the *type* of disorder experienced (see Goodman et al., 2018), as some symptoms may be more antithetical to experiences of wellbeing than others. For example, feelings of hopelessness, anhedonia, and generalised withdrawal in depression—at least when severe—could be seen as directly contradictory to a vital and thriving daily life. In contrast, individuals who exhibit focal distress or avoidance with limited situational breadth (e.g., some anxiety disorders) may retain the capacity for flourishing wellbeing more generally.

Few studies have empirically tested the dual-continua model in clinical populations, with mixed findings thus far. In support of the model, 15 % of participants in a nationally representative sample of Americans with a current mental illness diagnosis also displayed moderate or high wellbeing (Keyes, 2005). Other studies have used factor analysis to examine whether distress and wellbeing load on one factor or separate into two factors in clinical samples. Franken et al. (2018) found optimal fit for a two-factor model across multiple diagnostic groups (i.e., personality, developmental, anxiety, and mood disorders), though correlations between factors were strong (*r* = −0.61 to −0.79). Similarly, van Erp Taalman Kip and Hutschemaekers’ (2018) factor analysis identified a two-factor distress-wellbeing solution, but canonical correlation analysis supported a model with only one common canonical factor. Finally, a recent meta-analysis of the association between distress and wellbeing found that clinical status was not a significant moderator, although this result should be interpreted with caution given only 5 % of the included samples were clinical (Sgherza et al., 2025).

### 1.2. Momentary distress and wellbeing states in daily life

Existing evidence for the dual-continua model largely relies on cross-sectional data, or longitudinal data with a small number of measurements over relatively large time periods. Importantly, cross-sectional data can only be used to assess *between-person* associations (Curran and Bauer, 2011). That is, on average, do individuals who generally experience higher levels of distress symptoms than other people, also generally experience lower wellbeing than other people? Conversely, *within-person* associations test relationships between two or more variables within the same individual (Curran and Bauer, 2011). Analysis at the within-person level is highly relevant to the dual-continua model, given its potential to guide therapeutic decisions around whether to target intraindividual change in wellbeing, distress, or both (Iasiello et al., 2020), and evidence that distress and wellbeing often fluctuate over hours and days (Fried et al., 2022; Houben et al., 2015; Schoevers et al., 2021). Thus, we might ask, if an individual’s distress increases from one moment to another, does their wellbeing also decrease over the same interval? In psychology – where mechanisms occurring *within the individual* are often of key interest – it is crucial to distinguish between-person and within-person effects (Curran and Bauer, 2011), as associations often vary across levels (Hoffman and Stawski, 2009). Accordingly, understanding how distinctly distress and wellbeing operate within individuals provides direct information about their change processes, leading to more accurate clinical recommendations.

Ecological momentary assessment (EMA) involves repeatedly measuring states over short time periods, in naturalistic environments, to capture real-time fluctuations in individual experiences (Shiffman et al., 2008). When each participant has multiple reports, the data contain information about both between- and within-person associations (Curran and Bauer, 2011), making EMA an essential tool for isolating and testing within-person relationships. Additionally, EMA methods yield benefits such as reducing recall biases associated with retrospective/trait reports, and stronger ecological validity due to the measurement of experiences during one’s daily activities (Shiffman et al., 2008).

To our knowledge, only one previous study has investigated the dual-continua model within-persons using an EMA design. Kraiss et al. (2022) measured momentary distress and wellbeing three times per day for two weeks in 25 university students, finding moderate negative associations between distress and wellbeing at both the between- (β = −0.36, marginal *R*^2^ = 0.15) and within-person (β = −0.43, marginal *R*^2^ = 0.18) levels. Though findings were supportive of the dual-continua model, people also differed substantially in the degree of associations between the constructs. Notably, Kraiss et al. (2022) used a very small, unselected sample, so the generalisability and robustness of findings require further testing and cautious interpretation. Additionally, there was likely limited representation of individuals with clinically significant symptoms, and no examination of moderators (e.g., diagnostic status) that might account for the observed individual differences in the distress-wellbeing association.

### 1.3. The present study

The present study has two primary aims. The first is to test the within- and between-person distress-wellbeing association in a large community sample. The second aim is to investigate whether having a current psychological diagnosis (i.e., depression and anxiety disorders) moderates these within-person distress-wellbeing associations. We used an EMA design to assess current naturalistic experiences of distress (indicated via dysphoria symptoms) and wellbeing over a 7-day period. Wellbeing was assessed as both *hedonic* wellbeing (i.e., feeling good, or high positive affect and low negative affect; Diener, 1984) and *eudaimonic* wellbeing (i.e., functioning well, including life purpose and vitality; Ryff, 1989). Hedonic and eudaimonic wellbeing represent related yet distinct dimensions of wellbeing (Joshanloo, 2016) that relate differently to different types of psychopathology (Goodman et al., 2018).

Given previous findings in cross-sectional studies and one daily life study, we predict that distress and wellbeing will be inversely related at the within-person level, though not so strongly as to be redundant (e.g., *r* < −0.80; Aim 1). Our investigation of moderation (Aim 2) is relatively exploratory given no prior tests of the research question. However, based on theoretical justification and findings of significant between-subject heterogeneity in the strength of within-person distress-well-being association (Kraiss et al., 2022), we predict a small to medium moderation effect such that a stronger negative within-person association will be observed between distress and hedonic and eudaimonic wellbeing for those with a clinical diagnosis. Given the theoretical possibility that the strength of the distress-wellbeing association may differ by disorder type, we also tested moderation separately for depressive disorders and anxiety disorders.

## 2. Method

### 2.1. Participants

Secondary data analysis was conducted on data collected from 2017 to 2019, which aimed to assess the impact of decentering on daily emotional and cognitive outcomes (see Naragon-Gainey et al., 2023 for findings from the larger study). The sample consisted of 379 community members aged 18 to 65 from the Greater Buffalo area, New York, U.S.A. Participants were recruited through online advertisements (e.g., Craigslist), newspapers, and flyers in public spaces (e.g., cafes, universities, mental health clinics). Individuals seeking or receiving psychological treatment were over-sampled to increase the number of participants experiencing clinically-significant symptoms. Individuals were excluded if they did not speak English, and/or were diagnosed with untreated conditions (e.g., dementia, schizophrenia, or cognitive impairment) that may have caused differences in baseline cognitive tasks (not analysed in this study). No other published manuscripts from this dataset^1^ report this set of variables, or moderation of the dysphoriawellbeing association.

The final analysed sample included participants who completed the baseline and EMA portions. Twenty-three people who completed the baseline did not enrol into the EMA portion, and another 11 enrolled in but did not complete the EMA portion (submitted fewer than 30 % of valid EMA reports).^2^ The final sample included 345 participants (67.0 % female; age: M = 34.5 years, SD = 14.0) with racially diverse backgrounds (66.4 % White, 13.6 % Black or African American, 12.8 % Asian, 6.7 % more than one race, and 0.6 % Native American or Alaska Native). About half of the sample (49.6 %) reported currently receiving therapy or taking psychiatric medication. Nearly half (42.6 %) of the sample met criteria for at least one current disorder, with 12.0 % meeting criteria for major or persistent depressive disorder and 35.7 % meeting criteria for one or more anxiety disorders (i.e., panic disorder, social anxiety disorder, generalised anxiety disorder). Other demographic and diagnostic characteristics of this sample are presented in Table 1.

### 2.2. Procedure

Potential participants were screened via email or phone. Eligible participants provided informed consent and attended a 3 to 4-hour baseline appointment in the lab where they completed a clinical interview with trained graduate students. Participants also completed cognitive computer tasks, self-report measures, and a heart rate variability measurement (not analysed herein). Participants were paid $50 USD for completing the baseline portion.

Participants were invited to enrol in the EMA phase after completing the baseline assessment tasks. Upon consenting, a research assistant demonstrated how to complete EMA surveys and responded to participant questions. The EMA phase commenced within the next 4 days, during which participants received six brief surveys daily for 7 consecutive days on a smartphone. Links to the Qualtrics surveys were sent via text message through the SurveySignal system (Hofmann and Patel, 2015). Surveys were sent between 9 am and 9 pm, with one survey sent randomly within six two-hour blocks, constrained by a required minimum of 60 min between surveys. Each survey expired after 30 min, and a reminder was sent after 20 min. Incoming survey data were screened daily to monitor adherence and we contacted participants via email or phone if issues arose. Participants were compensated $1.50 USD for each survey completed during the EMA phase (i.e., $78 USD maximum) and an additional $15 USD bonus if fewer than nine surveys were missed. As an additional incentive, participants were entered into a raffle for each completed survey, increasing their chances of receiving one of four iPads. This study was approved by the Institutional Review Board at the University at Buffalo.

### 2.3. Measures

#### 2.3.1. Anxiety and related disorders interview schedule for DSM-5 (ADIS-5)

The ADIS-5 (Brown and Barlow, 2014) is a semi-structured interview designed to reliably diagnose and dimensionally assess a range of internalising disorders in accordance with the DSM-5 diagnostic criteria. Severity of each diagnostic criterion and disorder is rated on an 8-point scale (clinical severity rating; CSR) from none to very severe, and categorical DSM-5 diagnoses can be derived from these ratings. The following disorders were assessed: panic, social anxiety, generalised anxiety, obsessive compulsive, post-traumatic stress, major depressive, persistent depressive, bipolar, and cyclothymia. The presence of one or more of these diagnoses indicated clinical status in the moderation analyses, with further analyses conducted for depression (major depression, persistent depression) or anxiety (panic, social anxiety, generalised anxiety disorder) diagnoses. Interviews were conducted by trained graduate students, with supervision via regular diagnostic consensus meetings with a clinical psychologist. Interrater reliability was assessed in a subset of the sample (*n* = 68; 18 % of the full sample) via review of the interview audio recording by an independent second rater. For disorders in this subsample that had more than two cases of diagnoses, kappas ranged from 0.39 to 0.85 (i.e., fair to excellent agreement; mean kappa = 0.60).

Diagnostic status was the moderator in the primary moderation analyses to distinguish individuals with and without clinically significant levels of distress and/or impairment, as this distinction may be key to differences in distress-wellbeing associations. However, we also conducted robustness checks using a range of plausible dimensional operationalisations of clinical severity: mean CSR (i.e., average severity across all disorders to capture the breadth of symptoms), maximum CSR (i.e., greatest severity for any single disorder to capture the highest intensity of symptoms), and number of diagnoses (i.e., degree of comorbidity).

#### 2.3.2. EMA surveys

In each survey, participants were presented with the following instructions: *What follows is a series of questions that asks about your recent experiences. Please answer these with respect to the past 30* min*, including this moment*. The survey instructions and time frame were designed to prevent responses solely based on participant experiences exactly in the moment (i.e., while completing the survey) and to capture experiences with variables that tend to occur periodically rather than constantly (e. g., distress, rumination). Participants responded on a 5-point Likert scale from 1 (*very slightly or not at all*) to 5 (*extremely*). EMA surveys included 29 items; constructs assessed but not analysed here were emotion regulation strategies, mindfulness, decentering, meta-awareness, self-control, and most bothersome symptoms. We analysed the following composites: distress (operationalised via four dysphoria items: I had little interest in my usual hobbies and activities; I felt inadequate; I felt discouraged about things; I felt depressed), eight hedonic wellbeing items (upset, sad, afraid/anxious, irritable, all of which were reverse-keyed; active, interested, excited, strong), and two eudaimonic well-being items (How much meaning have you felt in your life recently?; To what extent have you felt alive and vital recently?). The distress, hedonic wellbeing, and “vitality” eudaimonic wellbeing items were taken from previous EMA studies (e.g., Breines et al., 2008; Naragon-Gainey and DeMarree, 2017), and the “meaning” eudaimonic wellbeing item was adapted from a state measure (Lambert et al., 2013).

The EMA composites showed acceptable internal consistency at the within-person and between-person levels (see Geldhof et al., 2014 for formulas and syntax): Distress omega within = 0.78, between = 0.95, Hedonic Wellbeing omega within = 0.80, between = 0.91, and Eudaimonic Wellbeing correlation [two items only] within *r* = 0.56, between *r* = 0.89. Between-person variance of the EMA composites for each variable showed moderate to strong correlations with their relevant baseline trait measures, supporting their validity (*r*s = 0.46 to 0.69, *p*s < 0.001; see Appendix A in supplementary material of Naragon-Gainey et al., 2023 for more details). Finally, EMA survey responses were considered invalid if they were completed outside the 30-min expiry window, or more than half of the items were answered in less than one second – a threshold supported by previous studies (e.g., Jaso et al., 2022). Overall, 11,954 out of 14,490 surveys (82.5 %, SD = 14.7 %) were considered valid.

### 2.4. Data analysis

Multilevel structural equation modelling (MSEM) in Mplus Version 8.3 (Muthén & Muthén, 1998–2017) was used to assess multilevel correlations at the within-person level (Aim 1), and test whether clinical status or severity moderates the association between distress and well-being (with separate models for hedonic and eudaimonic wellbeing) in daily life (Aim 2). MSEM creates orthogonal latent variables for between- and within-person variance of each EMA variable whereby the covariance between levels is set to zero, ensuring that the effects at each level are completely independent. As such, the within-person latent variable is implicitly person-mean centered (Preacher et al., 2010). We used Bayesian estimation to account for missing data and any non-normality in variable distributions. Standardised parameter estimates are reported with 95 % credibility intervals and were estimated using the STDYX command in Mplus which standardises predictor and outcome variables based on their respective variance at the relevant level. Multilevel correlations were interpreted in accordance with Cohen’s (1988) guidelines (i.e., |*r*| = 0.1, 0.3, 0.5 as weak, moderate, and strong, respectively). In accordance with previous studies (e.g, Kraiss et al., 2024) an effect size of effect heterogeneity (EH) was computed to quantify heterogeneity in random slopes. This was computed by dividing the standard deviation of the random slope by the average fixed effect of that slope. EH values >0.25 indicate significant heterogeneity (Bolger and Laurenceau, 2013).

Cross-level interactions were specified, wherein clinical status or severity was tested as a moderator of individual differences in the within-person distress-wellbeing associations. Separate models were run using Any Diagnosis, Anxiety, and Depression as between-person moderators of the within-person distress-hedonic and distress-eudaimonic wellbeing associations. Dimensional moderators (i.e., maximum severity, mean severity, number of diagnoses) were grand-mean centered. The temporal ordering of each report (i.e., 1–42) was included as a within-person covariate to account for linear trends over time. Significant interactions were probed via simple slopes analysis, as specified by Bauer and Curran (2005), which allows for analysis of how within-person relationships (e.g., distress and wellbeing) vary at levels of the between-person moderators (e.g., diagnostic status). Simple slopes for significant moderators were assessed at presence and absence of diagnoses for the primary analyses, and at low (1.5 standard deviations below mean), medium (mean), and high (1.5 standard deviations above mean) levels of dimensional moderators in our robustness checks.

## 3. Results

### 3.1. Descriptive statistics

Table 2 shows the between-person means (within-person means are set to zero by default in Mplus), within- and between-person standard deviations, intraclass correlations, and within- and between-person correlations among Distress, Hedonic and Eudaimonic Wellbeing, and indices of clinical status and severity (Any Disorder, Anxiety, and Depression). All correlations were statistically significant and in the expected direction. For the clinical subsample with one or more depression or anxiety disorders, CSRs indicated “moderate” to “severe” symptoms on average (M = 5.18, SD = 1.15, range = 3–8). In contrast, clinical severity for non-diagnosed participants was less than mild, on average (M = 1.80, SD = 1.00, range = 0–3).

### 3.2. Aim 1: distress-wellbeing associations

At the within-person level, Hedonic Wellbeing and Distress (*r* = −0.60) were strongly correlated, whereas a moderate within-person correlation was observed between Eudaimonic Wellbeing and Distress (*r* = −0.37). However, there was substantial heterogeneity in the distress-hedonic wellbeing associations (EH = 1.04) and distress-eudaimonic wellbeing association (EH = 0.54), suggesting significant variability in associations across individuals. At the between-person level, having Any Disorder, Depression, or Anxiety was moderately correlated with daily life Distress, Hedonic Wellbeing, and Eudaimonic Wellbeing (|*r*s| = 0.31 to 0.43). Intraclass correlations indicated that 48–61 % of the variance in Distress and Wellbeing was due to individual differences, whereas 39–52 % of the variance was due to within-person fluctuations and error variance.

### 3.3. Aim 2: moderating effect of clinical diagnostic status and severity

Results of multilevel regression models are shown in Tables 3–5. In all models, standardised regression coefficients indicated that Distress was significantly associated with lower Hedonic Wellbeing and Eudaimonic Wellbeing at a given report (within-persons), and clinical status/severity was significantly associated with lower Hedonic Wellbeing and Eudaimonic Wellbeing throughout the study (between-persons). Thus, main effects were in the expected direction. Levels of reported Hedonic and Eudaimonic Wellbeing significantly declined throughout the study in all models.

Primary moderation analyses focused on clinical diagnostic status as the moderator, with separate analyses for the presence of Any Disorder, an Anxiety diagnosis and a Depression diagnosis (Table 3). Overall clinical status, as indexed by Any Disorder diagnosis, did not moderate the association between momentary Distress and Hedonic Wellbeing (β = 0.02, CI: −0.08, 0.10) or Eudaimonic Wellbeing (β = 0.09, CI: −0.03, 0.20), nor did any Anxiety diagnosis (Hedonic Wellbeing β = 0.03, [CI: −0.07, 0.11], Eudaimonic Wellbeing β = 0.11, [CI: −0.01, 0.22]). However, Depression diagnosis did significantly moderate the association of Distress with Hedonic Wellbeing (β = 0.12, CI: 0.03, 0.21) and Eudaimonic Wellbeing (β = 0.14, CI: 0.04, 0.24). Contrary to our predictions, simple slope analysis showed that Depression diagnosis was associated with a *weaker* relationship of Distress with both Hedonic and Eudaimonic Wellbeing (Fig. 1, Panels A–B). That is, for individuals with a depressive disorder, Distress and both components of wellbeing appear to operate in a *less* bipolar pattern, compared to those without a depressive disorder.

### 3.4. Robustness checks

Robustness checks assessed whether moderation results held when clinical severity was assessed dimensionally, using mean severity across disorders, maximum severity of any single disorder, and number of diagnoses (see Tables 4 and 5 for full results). As in the primary moderation analyses, clinical severity (mean or maximum) for any disorder or anxiety disorders did not moderate the within-person association between Distress and either type of wellbeing. Similarly, the number of diagnoses was not a significant moderator. However, for depressive disorders, mean clinical severity significantly moderated the relationship of Distress with Hedonic Wellbeing (β = 0.13, CI: 0.04, 0.23) and Eudaimonic Wellbeing (β = 0.13, CI: 0.03, 0.23), whereas maximum clinical severity significantly moderated the relationship between Distress and Hedonic Wellbeing (β = 0.11, CI: 0.02, 0.20), but not Eudaimonic Wellbeing (β = 0.09, CI: −0.01, 0.18). For significant interactions, simple slopes analysis once again revealed that higher Depression severity was associated with a *weaker* relationship between Distress and Hedonic and Eudaimonic Wellbeing (Fig. 1; Panels C–E), consistent with the pattern of interaction in the primary moderation analyses for depression status.

## 4. Discussion

The present study tested the dual-continua model of mental health in clinical and non-clinical populations in daily life. Specifically, we examined the association of distress and wellbeing in daily life, as well as clinical status as a moderator of this within-person relationship.

### 4.1. The dual-continua model in daily life states

In line with our hypothesis, distress and wellbeing (both hedonic and eudaimonic) were found to operate as inversely related, yet distinct, constructs when assessed as momentary states in daily life. The moderate to strong negative within-person associations observed between distress and wellbeing suggest that when individuals report more distress than is typical for them, they also tend to report lower hedonic and eudaimonic wellbeing at the same timepoint. However, this relationship was not strong enough to suggest distress and wellbeing operate on a single bipolar continuum. The strength of the associations observed herein is consistent with the moderate association observed by Kraiss et al. (2022), whose assessment of wellbeing most closely aligned with eudaimonic wellbeing. Taken together, these findings indicate that distress and wellbeing are moderately related when assessed as momentary states in daily life, suggesting evidence for the dual-continua model at the within-person level. However, like Kraiss et al. (2022), we also found substantial heterogeneity in the magnitude (and even sign) of the distress-wellbeing association across individuals, suggesting a need for further investigation of variables that might predict these differences.

Previous research suggests investigation of the dual-continua model may be most fruitful at the subdomain level, where a narrower focus on distinct subdomains of distress/wellbeing may yield the most meaningful insights (Mason Stephens et al., 2023). Although a few studies have separately considered hedonic and eudiamonic wellbeing in relation to the dual-continua model (e.g., Franken et al., 2018; Lamers et al., 2011; Weich et al., 2011), the present study is the first to provide fine-grained analysis at the within-person level. Our finding that the distress-hedonic wellbeing (vs distress-eudaimonic wellbeing) association was stronger in daily life is consistent with the antonymous definitions of distress and hedonic wellbeing, and the generally high levels of anhedonia observed among clinically distressed individuals (Taylor et al., 2022; Whitton and Pizzagalli, 2022).

### 4.2. Moderating effect of clinical diagnostic status and severity

Contrary to our hypotheses that clinical status would be associated with a stronger within-person relationship between distress and hedonic and eudaimonic wellbeing, being diagnosed with any disorder or an anxiety disorder was not associated with individual differences in the distress-wellbeing association. This null result held when examining symptoms dimensionally in robustness check analyses. Thus, though clinical populations typically experience more distress and lower levels of hedonic and eudaimonic wellbeing than the general population (Goodman et al., 2018; Keyes, 2007), our results indicate that distress and wellbeing remain distinct and only moderately associated within clinical populations generally and anxiety specifically. Accordingly, these findings suggest that a comprehensive assessment of mental health in daily life requires consideration of both distress and wellbeing, regardless of the individual’s clinical diagnostic status. That is, measuring one’s distress does not provide a full understanding of their hedonic and/or eudaimonic wellbeing at the same time-point.

Opposite to our hypothesised pattern of moderation, depression diagnosis and severity were associated with a significantly *weaker* relationship between distress and both hedonic and eudaimonic well-being. That is, distress and wellbeing were more distinct for people with depression than for people without depression, suggesting that the dual-continua model applies to this subgroup even more clearly than the general population. Though unexpected, there are plausible post hoc explanations for this finding. According to the previously described *dynamic model of affect*, additional cognitive load triggered by heightened distress causes affective states to become less distinguishable. Although this would theoretically strengthen the negative distress-wellbeing association in clinical populations, such an effect may not apply to those with chronic states of depression, in part due to adaptation to chronically elevated negative emotions/low positive emotions (e. g., Frederick and Loewenstein, 1999; Helson, 1964). That is, in contrast to the persistent undercurrent of depression experienced, moments of positive emotion (hedonic wellbeing) or meaning and purpose (eudaimonic wellbeing) may become *more* salient for those with depression, leading to the possibility for states of distress and wellbeing to become less closely yoked.

This idea relates to the established *mood brightening effect*, or a steeper momentary increase in positive affect following positive events in daily life for people with depression (compared to other disorders or healthy controls; Khazanov et al., 2019; Peeters et al., 2003). The mood brightening effect does not inherently provide evidence for the dual-continua model, as it does not exclude the possibility that momentary increases in positive affect are reciprocated with an equal and opposite decrease in distress. However, taken with our finding that those with depression display a *weaker* within-person distress-wellbeing association, these findings suggest that momentary increases in wellbeing are not necessarily reciprocated with decreased distress in those with depression. Our results relating to eudaimonic wellbeing suggest that a comparable effect may extend to positive states of vitality, meaning and purpose – concepts less closely related to positive affectivity (Huta and Ryan, 2010).

Of note, it is also possible that our recruitment method – which required participant initiative to contact the researchers and complete screening – and the monetary incentives may have been less motivating to individuals with severe depression, given associated reductions in neural reward systems (Höflich et al., 2018). If so, people with depression in our sample may systematically differ from the population of depressed individuals. Accordingly, it will be important to determine the robustness and replicability of these results in future studies conducted in depression treatment settings.

### 4.3. Clinical implications

Overall, the observed independence between distress and wellbeing in daily life among clinical participants supports the idea that wellbeing should be measured and targeted within clinical populations (Craske et al., 2016; Fava et al., 2017; Wood and Tarrier, 2010). Though current psychotherapies contain elements designed to facilitate increased positive experiences through mechanisms such as response-contingent positive reinforcement (e.g., behavioural activation), such interventions do not always translate to increased *positive* mental health as expected (Craske et al., 2019; Moore et al., 2013).

Consequently, some interventions are specifically aimed at promoting positive states of hedonic wellbeing (e.g., Positive Affect Treatment [Craske et al., 2016]) and eudaimonic wellbeing (e.g., Wellbeing Therapy [Fava and Ruini, 2003]) in clinical populations. Research suggests such interventions can be more effective than traditional distress-focused interventions alone in increasing hedonic wellbeing (Craske et al., 2019) and improving depression and anxiety recovery and relapse rates (Fava et al., 1998; Fava et al., 2004). Although evidence for the clinical utility of wellbeing therapies is beyond the scope of the present investigation, our findings support the notion that overall mental health (low distress paired with high wellbeing; Keyes, 2005) should not be an assumed by-product of distress reduction. Furthermore, our findings provide foundation for further research aimed at optimising the utility of wellbeing-focused approaches in clinical populations – where lower levels of hedonic and eudaimonic wellbeing are common (Brandel et al., 2017; Goodman et al., 2018).

### 4.4. Strengths, limitations, and future directions

The intensive longitudinal design used is a notable study strength. EMA designs allow for repeated sharp recollection of recent experiences, increasing confidence in the ecological validity of findings (Shiffman et al., 2008), and allowing for observation of within-person effects that are unattainable via retrospective recall methods. Further, retrospective recall designs may yield an inflated negative wellbeing-distress association in clinical populations due to negative recall biases in those with internalising symptoms (Colombo et al., 2019). Despite the many utilities in using intensive longitudinal designs, the results obtained are correlational, thus no causal conclusions can be drawn.

A number of limitations should be considered. First, our exploration of multiple outcomes in an area with minimal prior research raises the possibility of Type 1 errors, and so results should be considered tentative until they are replicated in independent samples with different clinical features. Importantly, interpretation of findings related to the dual-continua model are limited to the conceptualisation and operationalisation of distress and wellbeing used herein, and these are often inconsistent across studies (Iasiello et al., 2020). We used a composite of positive and negative affect items to assess hedonic wellbeing. While this approach has been used previously (e.g., Breines et al., 2008; Lamers et al., 2011), some items share close conceptual connection to items assessing distress (dysphoria). For example, distress items such as “I felt depressed”, are conceptually antithetical to reverse coded hedonic wellbeing items such as “upset” and “sad”. The antonymous nature of these items likely inflated the strong within-person associations observed between hedonic wellbeing and distress.

In addition, the distress items used here, while highly correlated with general anxiety and depression, are more reflective of depression symptoms than anxiety and therefore may not fully capture general distress. Additionally, we did not assess all possible psychopathologies, instead focusing on internalising disorders (i.e., depression and anxiety). Accordingly, the results may not generalise to people with extreme clinical severity, varying disease course, or other types of disorders. As previously mentioned, the self-motivated recruitment methods and monetary incentives may have also influenced the generalisability of results, particularly related to those with severe depression. Furthermore, some undiagnosed participants in the present study may exhibit other (unassessed) types of psychopathologies.

Finally, the generalisability of results is also limited by use of a primarily female and White sample living in a Western country. Importantly, Eastern cultures tend to exhibit greater emotional complexity (the co-occurrence of positive and negative emotions) due to increased dialectical thinking, or the acceptance of seeming contradictions (Luttrell et al., 2022; Spencer-Rodgers et al., 2010). Consequently, we might expect the distinction between momentary distress and wellbeing to be more pronounced in non-Western cultures.

To increase confidence in the present findings, future research should seek to replicate these results using different (and diverse) measures of distress and wellbeing in various clinical populations. In clinical settings, arguably the most important question relating to the dual-continua model remains whether the cultivation of wellbeing is possible given the individual’s specific set of distress symptoms. Accordingly, further nuance could be attained by systematically investigating the relationships between more narrow symptoms of distress (e. g., anhedonia, mania, avoidance) and wellbeing (e.g., hedonic, eudaimonic, social) in various clinical populations. Such an enquiry could aid efforts to optimally apply wellbeing-promotion/distress-reduction in clinical populations with diverse presentations.

### 4.5. Conclusion

Overall, the present study provides further evidence that the dual-continua model holds true at the within-person level, and further, extends this evidence to those with a mental illness – a population where previous evidence for the model is less convincing (Iasiello et al., 2020). Our results indicate that, even for individuals with a current internalising disorder, experiences of distress and wellbeing in daily life remain somewhat distinct. If replicated, these findings suggest that the theory and implications of the dual-continua model may be equally applicable to clinical populations.

## Figures and Tables

**Fig. 1.**
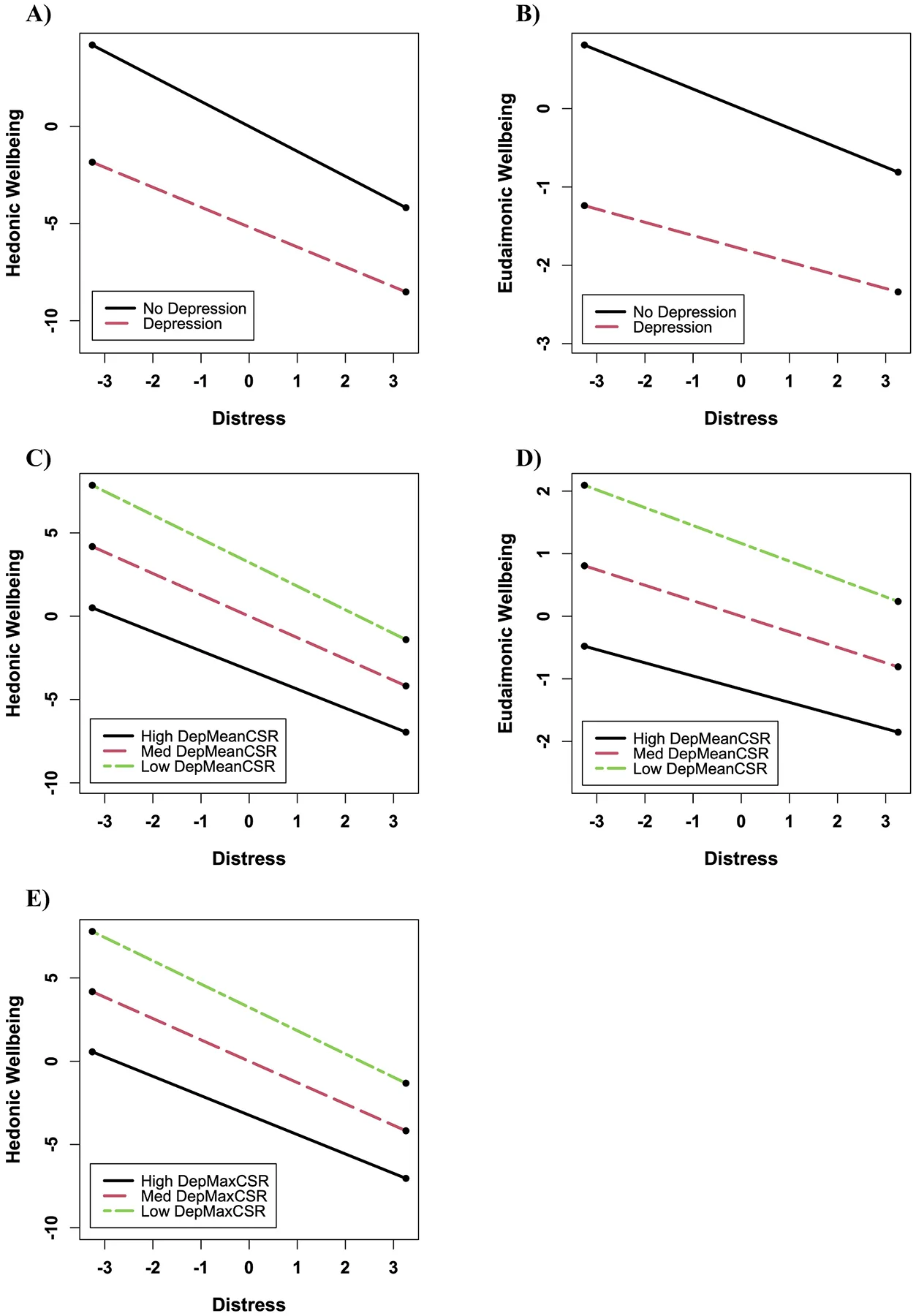
Simple slope analysis for the significant within-person moderating effects of depression diagnosis (Panels A–B) and severity (C–E) on hedonic and eudaimonic wellbeing.

**Table 1:** Demographic characteristics of the final sample.

Characteristic	Percentage of sample
Gender	
Female	67.0
Male	31.6
Other	1.4
Race	
White	66.4
Black or African American	13.6
Asian	12.8
More than one race	6.7
Native American or Alaska Native	0.6
Employment	
Employed part-time	33.9
Employed full-time	24.9
Unemployed	24.9
Full-time student	31.9
Part-time student	6.1
Working and student (full or part time)	19.0
Gross household income (USD)	
Less than $10,000	30.5
$10,000–$20,000	16.5
$20,000–$40,000	22.6
$40,000–$60,000	11.0
$60,000–80,000	6.1
More than $80,000	13.4
Marital status	
Single	55.7
Married	20.6
Single but cohabitating with a partner	12.8
Divorced	9.9
Widowed	1.2
Diagnoses	
One or more emotional disorder	42.6
Any depression diagnosis	12.0
Any anxiety diagnosis	35.7
Social anxiety disorder	25.8
Generalised anxiety disorder	20.6
Panic disorder	4.4
Major depressive disorder	6.5
Persistent depressive disorder	6.5

*Note*. Disorders were assessed with the ADIS-5. It was possible for participants to meet criteria for more than one diagnosis.

**Table 2:** Descriptive statistics and correlations.

	SD_W_	M_B_	SD_B_	1	2	3	4	5	6	7	8	9	10	11	12
1. Distress	2.17	6.91	2.72	(0.61)	−0.37	−0.60	–	–	–	–	–	–	–	–	–
2. Eudaimonic wellbeing	1.39	5.46	1.66	−0.55	(0.59)	0.61	–	–	–	–	–	–	–	–	–
3. Hedonic wellbeing	4.40	26.91	4.19	−0.76	0.83	(0.48)	–	–	–	–	–	–	–	–	–
4. Any diagnosis		0.43	0.49	0.43	−0.33	−0.43	–	–	–	–	–	–	–	–	–
5. Depression diagnosis		0.13	0.34	0.43	−0.32	−0.39	0.43	–	–	–	–	–	–	–	–
6. Anxiety diagnosis		0.36	0.48	0.37	−0.31	−0.40	0.86	0.24	–	–	–	–	–	–	–
7. Maximum disorder severity		3.15	1.93	0.47	−0.36	−0.46	0.84	0.40	0.78	–	–	–	–	–	–
8. Mean disorder severity		0.79	0.68	0.55	−0.39	−0.51	0.70	0.57	0.68	0.81	–	–	–	–	–
9. Maximum depression severity		1.24	1.81	0.53	−0.42	−0.49	0.48	0.83	0.37	0.54	0.72	–	–	–	–
10. Mean depression severity		0.73	1.21	0.52	−0.41	−0.48	0.45	0.82	0.37	0.52	0.76	0.95	–	–	–
11. Maximum anxiety severity		2.91	1.93	0.44	−0.35	−0.46	0.76	0.31	0.84	0.93	0.80	0.46	0.45	–	–
12. Mean anxiety severity		1.64	1.28	0.47	−0.34	−0.47	0.73	0.35	0.80	0.86	0.87	0.49	0.48	0.92	–
13. Number of diagnoses		0.77	1.26	0.53	−0.36	−0.48	0.78	0.57	0.77	0.76	0.91	0.63	0.67	0.82	0.74

*Note*. For EMA variables, within-person correlations are shown above the diagonal, between-person correlations are shown below the diagonal, and intraclass correlations are shown on the diagonal in parentheses. All correlations are significant at *p* < .001.

**Table 3:** Clinical diagnostic status as a cross-level moderator of the within-person associations of distress and wellbeing.

Clin diagnostic status	Any disorder		Depression		Anxiety	
	*β*	95 % CI	*β*	95 % CI	*β*	95 % CI
Outcome: hedonic						
Within-person						
Distress	−0.60*	(−0.62, −0.58)	−0.60*	(−0.62, −0.52)	−0.60*	(−0.62, −0.58)
Time trend	−0.06*	(−0.08, −0.04)	−0.06*	(−0.08, −0.03)	−0.06*	(−0.08, −0.04)
Between-person						
Clin status	−0.31*	(−0.37, −0.25)	−0.30*	(−0.39, −0.22)	−0.29*	(−0.35, −0.22)
Distress * clin status	0.02	(−0.08, 0.10)	0.12*	**(0.03, 0.21)**	0.03	(−0.07, 0.11)
Outcome: eudaimonic						
Within-person						
Distress	−0.38*	(−0.39, −0.32)	−0.37*	(−0.39, −0.35)	−0.38*	(−0.39, −0.32)
Time trend	−0.09*	(−0.10, −0.03)	−0.08*	(−0.10, −0.06)	−0.09*	(−0.10, −0.03)
Between-person						
Clin status	−0.24*	(−0.31, −0.17)	−0.25*	(−0.32, −0.17)	−0.22*	(−0.29, −0.15)
Distress * clin status	0.09	(−0.03, 0.20)	0.14*	**(0.04, 0.24)**	0.11	(−0.01, 0.22)

*Note*. CI = credibility interval; significant effects at *p* < .05 are highlighted with an asterisk and significant interactions in bold text. *β* = standardised regression coefficients.

**Table 4:** Clinical severity as a cross-level moderator of the within-person associations of distress and wellbeing.

Mean severity rating	Any disorder		Depression		Anxiety	
	*β*	95 % CI	*β*	95 % CI	*β*	95 % CI
Outcome: hedonic						
Within-person						
Distress	−0.60*	(−0.62, −0.58)	−0.60*	(−0.62, −0.52)	−0.60*	(−0.62, −0.58)
Time trend	−0.06*	(−0.08, −0.04)	−0.06*	(−0.08, −0.03)	−0.06*	(−0.08, −0.04)
Between-person						
Mean severity	−0.39*	(−0.45, −0.33)	−0.40*	(−0.45, −0.33)	−0.35*	(−0.41, −0.29)
Distress * mean severity	0.04	(−0.03, 0.13)	0.13*	**(0.04, 0.23)**	−0.00	(−0.08, 0.09)
Outcome: eudaimonic						
Within-person						
Distress	−0.38*	(−0.40, −0.32)	−0.37*	(−0.39, −0.35)	−0.38*	(−0.39, −0.32)
Time trend	−0.09*	(−0.10, −0.03)	−0.08*	(−0.10, −0.06)	−0.09*	(−0.10, −0.03)
Between-person						
Mean severity	−0.29*	(−0.35, −0.22)	−0.35*	(−0.41, −0.28)	−0.24*	(−0.31, −0.18)
Distress * mean severity	0.09	(−0.02, 0.18)	0.13*	**(0.03, 0.23)**	0.07	(−0.02, 0.17)
Max severity rating	Any disorder		Depression		Anxiety	
	*β*	95 % CI	*β*	95 % CI	*β*	95 % CI
Outcome: hedonic						
Within-person						
Distress	−0.60*	(−0.62, −0.58)	−0.60*	(−0.62, −0.52)	−0.60*	(−0.62, −0.58)
Time trend	−0.06*	(−0.08, −0.04)	−0.06*	(−0.08, −0.03)	−0.06*	(−0.08, −0.04)
Between-person						
Max severity	−0.35*	(−0.41, −0.29)	−0.39*	(−0.45, −0.32)	−0.34*	(−0.40, −0.28)
Distress * max severity	0.02	(−0.07, 0.12)	0.11*	**(0.02, 0.20)**	0.03	(−0.05, 0.12)
Outcome: eudaimonic						
Within-person						
Distress	−0.38*	(−0.39, −0.32)	−0.37*	(−0.39, −0.32)	−0.38*	(−0.39, −0.32)
Time trend	−0.09*	(−0.10, −0.03)	−0.08*	(−0.10, −0.03)	−0.09*	(−0.10, −0.03)
Between-person						
Max severity	−0.26*	(−0.32, −0.19)	−0.34*	(−0.40, −0.28)	−0.26*	(−0.32, −0.19)
Distress * max severity	0.09	(−0.03, 0.19)	0.09	(−0.01, 0.18)	0.09	(−0.01, 0.19)

Note. CI = credibility interval; significant effects at *p* < .05 are highlighted with an asterisk and significant interactions in bold text. *β* = standardised regression coefficients.

**Table 5:** Number of diagnoses at baseline as a cross-level moderator of the within-person relationship between distress and wellbeing.

	Distress	
	*β*	95 % CI
Outcome: hedonic		
Within-person		
Distress	−0.60*	(−0.62, −0.52)
Time trend	−0.07*	(−0.08, −0.02)
Between-person		
No. of diagnoses	−0.36*	(−0.43, −0.27)
Distress * no. of diagnoses	0.07	(0.00, 0.02)
Outcome: eudaimonic		
Within-person		
Distress	−0.37*	(−0.39, −0.32)
Time trend	−0.08*	(−0.10, −0.03)
Between-person		
No. of diagnoses	−0.26*	(−0.33, −0.19)
Distress * no. of diagnoses	0.10	(−0.01, 0.20)

Note. CI = credibility interval; significant effects at *p* < .05 are highlighted with an asterisk. *β* = standardised regression coefficients.

## Footnotes

1 Bylsma et al. (2024), Biehler and Naragon-Gainey (2022), DeMarree and Naragon-Gainey (2022), Naragon-Gainey et al. (2023), Park and Naragon-Gainey (2020) and Sgherza et al. (2022).

2 The final sample did not differ from the 34 participants excluded from EMA analyses in terms of sex, age, ethnicity, education level, marital status, and employment status (*p*s > 0.05). However, there were significant differences for race and household income between the two groups (*p*s < 0.05).
